# Coloration principles of the Great purple emperor butterfly (*Sasakia charonda*)

**DOI:** 10.1186/s40851-020-00164-6

**Published:** 2020-11-12

**Authors:** Doekele G. Stavenga, Hein L. Leertouwer, Kentaro Arikawa

**Affiliations:** 1 Surfaces and thin films, Zernike Institute for Advanced Materials, University of Groningen, Nijenborgh 4, NL-9747 AG Groningen, the Netherlands; 2 Department of Evolutionary Studies of Biosystems, Sokendai-Hayama, The Graduate University for Advanced Studies, Hayama, 240-0193 Japan

**Keywords:** Wing scales, Iridescence, Thin films, Structural coloration, Spectrophotometry, Scatterometry

## Abstract

The dorsal wings of male *Sasakia charonda* butterflies display a striking blue iridescent coloration, which is accentuated by white, orange-yellow and red spots, as well as by brown margins. The ventral wings also have a variegated, but more subdued, pattern. We investigated the optical basis of the various colors of intact wings as well as isolated wing scales by applying light and electron microscopy, imaging scatterometry and (micro)spectrophotometry. The prominent blue iridescence is due to scales with tightly packed, multilayered ridges that contain melanin pigment. The scales in the brown wing margins also contain melanin. Pigments extracted from the orange-yellow and red spots indicate the presence of 3-OH-kynurenine and ommochrome pigment. The scales in the white spots also have multilayered ridges but lack pigment. The lower lamina of the scales plays a so-far undervalued but often crucial role. Its thin-film properties color the majority of the ventral wing scales, which are unpigmented and have large windows. The lower lamina acting as a thin-film reflector generally contributes to the reflectance of the various scale types.

## Introduction

*Sasakia charonda*, the national butterfly of Japan since 1957, is locally known as *oh-murasaki*, or the great purple emperor. Indeed, the dorsal wings of the male display a striking, blue-purple central wing area, which is accentuated by a surrounding brown margin dotted with orange-yellow spots. A prominent red spot exists basally on the hindwings. The species is distinctly sexually dimorphic, as the dorsal wings of the (slightly larger) female lack the brilliant blue-purple iridescence of the male [3].

As holds universally for butterflies, the color pattern is created by a lattice of scales that covers the wings like tiles on a roof. The scale material, chitin, is structured into a thin, plate-like lower lamina and an elaborate upper lamina. The latter consists of numerous parallel ridges made up of narrow piles of partly overlapping lamellae that are connected by crossribs [5–7].

The scale structures scatter incident light, which, in the absence of pigment, results in white-colored scales. In the presence of pigments, the scattered light is filtered, dependent on the pigment’s absorption spectrum, thus resulting in a pigmentary (or chemical) color. The pigments expressed in the scales are rather characteristic for the various butterfly families [14]. For instance, pterins are the dominant pigments in Pieridae [2, 31], ommochromes and their precursor 3-OH-kynurenine color the wings of Nymphalidae [4, 10, 15, 19], and molecular compounds of kynurenine bound to N-β-alanyl-dopamine (NBAD) create the papiliochromes found in Papilionidae [16, 17, 27]. All butterfly families employ melanin in brown and black scales.

When the scales contain regular-arranged, nanosized structures, light interference creates a structural (or physical) color. Notably, the lower lamina, because of its thickness being on the order of 100–300 nm, always acts as a thin-film reflector [18, 22]. By adjusting the actual thickness, the color of the reflected light can be finely tuned [22, 30]. Another structural coloration is well known from the iconic *Morpho* butterflies; their scales have ridges folded into chitin-air multilayers, which create intensely blue wings [7, 28]. Further tuning of the scale color can occur by combining the structural coloration with wavelength-selective absorbing pigments [32, 33], but when the scale contains a high melanin concentration, its color is lost [25, 29].

Pigmentary coloration is generally diffuse and nondirectional, while structural coloration is often called metallic because the reflected light strongly depends on the directions of illumination and viewing. The two coloration mechanisms, which thus can be easily distinguished, are both applied in the wings of male *S. charonda*. The brown, yellow and red scales are found to be colored by pigments deposited in scales having the basic butterfly scale structure, where the ridges are made up of a few (at most 2–3) overlapping lamellae. By contrast, the blue-purple scales of the central wing area have enhanced ridges with up to seven overlapping lamellae, which thus create a multilayer causing a distinct iridescent coloration [11, 12].

Medina et al. [13] studied the spatial and spectral characteristics of the iridescent wings of *S. charonda*, but the applied spectral analysis focused on human colorimetry and thus was restricted to wavelengths above 400 nm. The visual system of *S. charonda* includes ultraviolet light, as the photoreceptors have peak wavelengths at ~ 340, 430 and 550 nm [9]; therefore, the wing reflectance spectra should also be measured in the short wavelength range.

Here, we present a more comprehensive analysis of the spectral properties of the variously colored wing areas together with the anatomy and spatial reflection characteristics of their scales. We confirm that the prominent blue wing coloration is caused by scales with multilayered ridges, but we find that the thin-film reflectors of the scales’ lower laminae, often combined with different pigments, importantly determine the coloration of *S. charonda*. The optical mechanisms determining butterfly coloration appear to be quite universally applicable to other nymphalid butterflies [8, 25].

## Materials and methods

### Specimens and photography

A mounted male *S. charonda charonda* (Japanese subspecies) of the Sokendai-Hayama butterfly collection was photographed with a Nikon D70 digital camera equipped with an F Micro-Nikkor macro objective (60 mm, f/2.8; Nikon, Tokyo, Japan). UV images were made by using a blacklight lamp and the camera’s red channel. We applied normally incident, obliquely incident and transmitted light. Anatomy was performed on another specimen of *S. c. charonda*. Additional specimens captured in China and Korea were purchased from a commercial supplier (The Bugmaniac). Close-up photographs of small wing areas and isolated scales of both the abwing (upper) side and adwing (under) side (i.e., facing the wing) were made with a Zeiss Universal microscope using a Zeiss Epiplan 16x/0.35 objective (Zeiss, Oberkochen, Germany).

### Spectrophotometry

Wing reflectance spectra were measured with a bifurcated probe connected to a halogen/deuterium light source and an Avantes AvaSpec-2048-2 CCD detector array spectrometer (Avantes, Apeldoorn, Netherlands). The angle of illumination was approximately normal with respect to the wing surface. A microspectrophotometer consisting of a Leitz Ortholux microscope and the Avantes spectrometer was used to measure the reflectance spectra of individual scales, which were removed from the wing and glued to the tip of pulled-glass pipettes. The reference for all reflectance measurements was a white diffuse standard (Avantes WS-2). The absorbance spectra of the pigment extracts, obtained by immersing small yellow and red wing areas in a solution of 50:1 methanol: 1 M hydrochloric acid, were measured with a setup consisting of the halogen/deuterium light source, optical fibers, and the detector array.

### Anatomy of the wing scales

To examine the scales’ anatomy, wing pieces were sputtered with platinum and observed with a JSM-6490LV scanning electron microscope (JEOL, Tokyo, Japan). For transmission electron microscopy, wing parts were prefixed in 2% paraformaldehyde and 2.5% glutaraldehyde in 0.1 mol l^− 1^ sodium cacodylate buffer (CB, pH 7.3) overnight at 4 °C. Embedding in Spurr was followed by dehydration with a graded series of acetone and infiltration with propylene oxide. Tissue sections with a thickness of 50 nm were observed with an H-7650 transmission electron microscope (Hitachi, Tokyo, Japan) equipped with a CCD camera (Quemesa, Seika Digital Image Corp., Tokyo, Japan), and the images were analyzed with iTEM software (Olympus, Tokyo, Japan).

### Imaging scatterometry

Imaging scatterometry was applied to visualize the far-field angular distribution of the light scattered from single scales glued at the end of pulled-glass micropipettes. The sample was positioned in the first focal point of the scatterometer’s ellipsoidal mirror, which collects light from a full hemisphere. A narrow aperture beam (5°) provided by a xenon lamp illuminated a small area of a scale (diameter ~ 13 μm). A piece of magnesium oxide served as a white diffuse reference object. Scatterogram images were acquired by an Olympus DP70 camera (for details, see [24]).

### Modeling of the reflectance spectra

The reflectance spectrum of the lower lamina of the scales was calculated for normally incident light using the classical Airy formulas for thin films [22, 23].

## Results

### Wing patterning and coloration

**Fig. 1:**
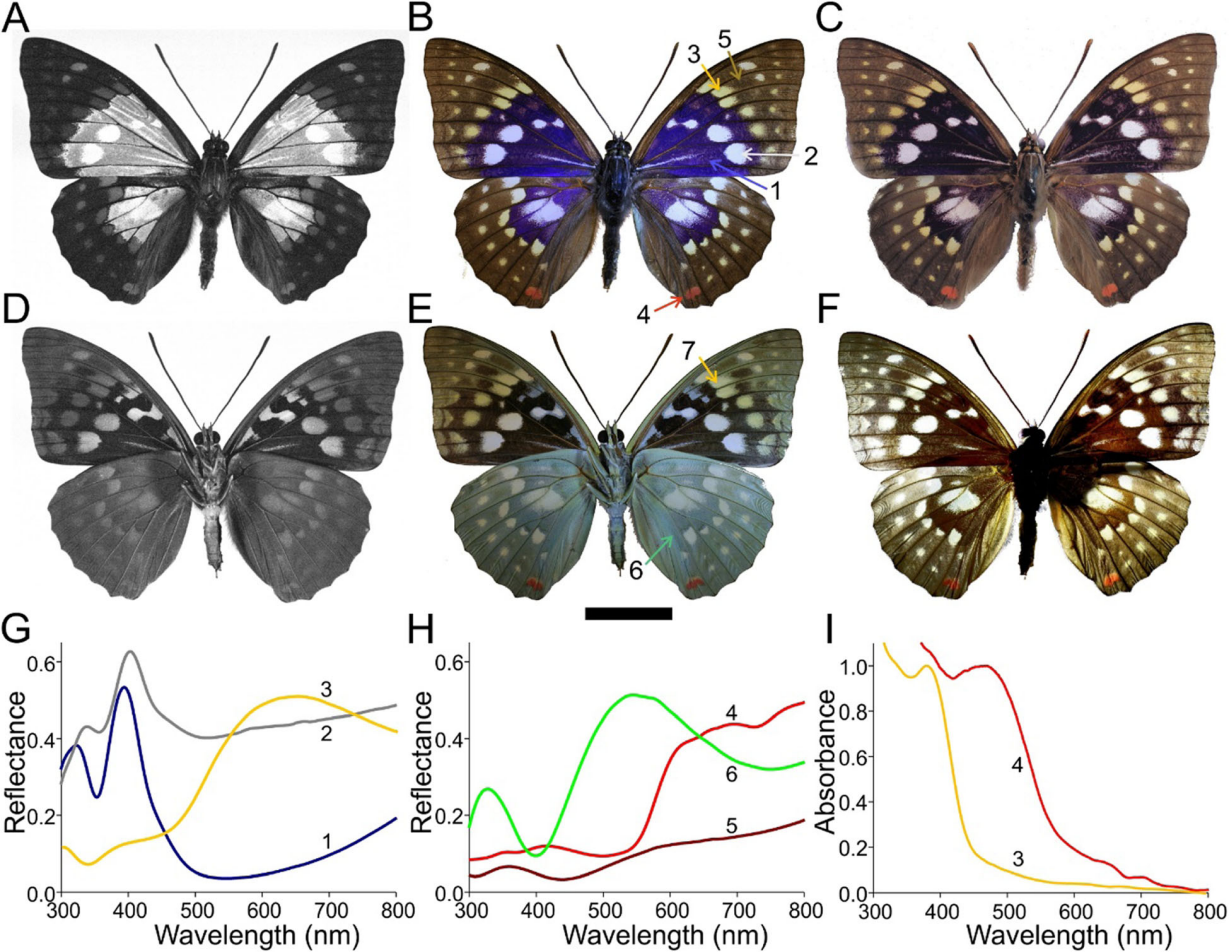
The great purple emperor butterfly, *Sasakia charonda* (male). **a**, **b**, **c**, **f** Dorsal views. **d**, **e** Ventral views. **a**, **d** UV images. **b**, **c**, **e**, **f** RGB images. **a**, **b**, **d**, **e** Normal illumination. **c** Oblique illumination (angle of incidence of ~ 45°). **f** Illumination from below, showing transmitted light. Scale bar: 2 cm. **g**, **h** Reflectance spectra measured with a bifurcated probe. **i** Absorbance spectra (normalized at the long-wavelength peak) of pigments extracted from wing pieces cut from an orange-yellow area on the forewing (#3) and from a red spot on the hindwing (#4). The numbers 1–6 in **g-i** correspond to the numbered areas in **b** and **e**; the number 7 in **e** corresponds to Fig. 3p-t

The dorsal wings of male *S. charonda* display a contrasting pattern created by a dark brown margin and cream to orange-yellow spots that surround a UV/blue-reflecting central area, which furthermore contains bright white spots (Fig. 1a, b). Upon oblique illumination with an angle of incidence of ~ 45° (Fig. 1c), the blue reflection of the central area (Fig. 1b, #1) vanishes, which demonstrates its iridescence. The reflectance spectra of the blue wing parts show a narrow band (bandwidth ~ 50 nm) peaking at ~ 400 nm (Fig. 1b, g, #1). Not surprisingly, the centrally located white spots show a high reflectance throughout the whole wavelength range, but an enhanced UV/blue reflectance peak also exists (Fig. 1b, g, #2). The orange-yellow patches adjacent to the central blue area have a broadband reflectance spectrum (Fig. 1b, g, #3), while the reflectance of the red spots of the dorsal hindwings is more restricted to longer wavelengths (Fig. 1b, h, #4). The reflectance of the brown margins is low throughout the whole wavelength range and slightly increases with increasing wavelength, characteristic of melanin-pigmented tissue; however, the minor peak in the UV spectrum suggests a structural contribution (Fig. 1b, h, #5).

The pattern on the ventral wings resembles that on the dorsal wings, but the strong blue coloration of the dorsal wings is absent. The ventral hindwings display a greenish shimmer, suggesting a structural basis (Fig. 1e, h, #6). The dark brown margins and the orange-yellow, red and white spots appear to be colocalized on both wing sides, which is confirmed when applying transillumination (Fig. 1f). This finding demonstrates that the white areas are transparent, i.e., unpigmented. The orange-yellow spots on the fore- and hindwings are also rather clear in transmitted light, which indicates that pigment absorption in the visible wavelength range is minor. The red spots on the hindwings are red in both epi- and transillumination, which means that absorption in the visible wavelength range is considerable.

To investigate the light-absorbing pigments more closely, we measured the absorbance spectra of the pigments extracted from the yellow and red spots (Fig. 1i, #3, 4). The obtained spectra show a high absorbance in the wavelength ranges where the reflectance is low (Fig. 1g, h, #3, 4). This finding indicates the prominent presence of 3-OH-kynurenine and ommochrome pigment in the yellow and red scales, respectively. The central and marginal wing areas are dark and light brown, respectively, or equivalently, the melanin pigment concentration gradually decreases from the butterfly’s body towards the periphery of its wings.

### Electron microscopy of the scale types

**Fig. 2:**
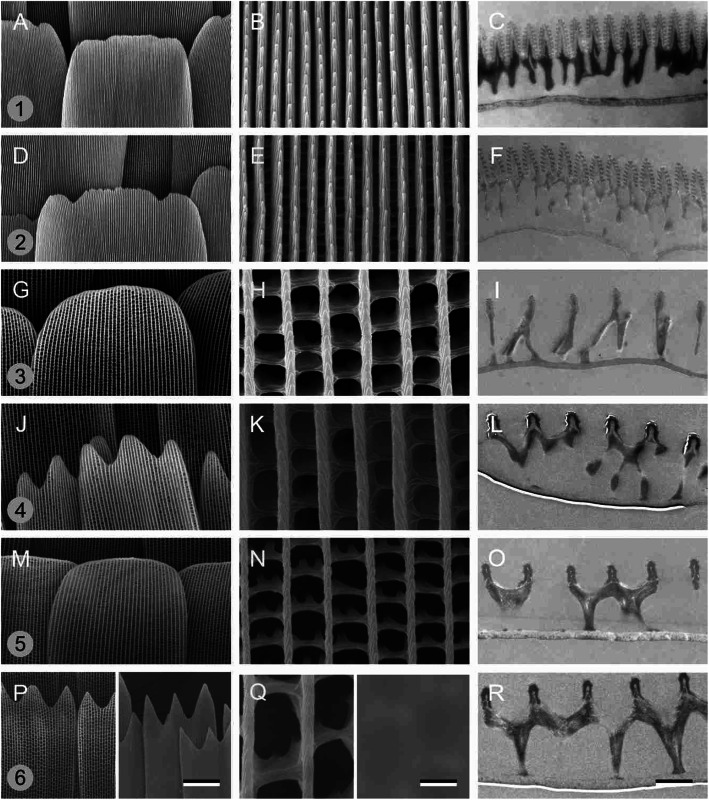
Electron micrographs of various wing scales. **a**-**c** Blue scales. **d**-**f** White scales. **g**-**i** Orange-yellow scales. **j**-**l** Red scales. **m**-**o** Brown scales. **a**-**o** Dorsal wing scales. **p**-**r** Greenish ventral wing scales. Left and middle columns: scanning electron microscopy (**p** and **q** left half: from the abwing side; right half: from the adwing side), right-hand column: transmission electron microscopy; scale bars: 20 μm (left column), 2 μm (middle and right columns). The numbers 1–6 in **a**, **d**, **g**, **j**, **m**, and **p** correspond to the numbered areas in Fig. 1b, e

The scales are not only colored by pigments, but their structure is certainly important as well (Fig. 2). Scanning electron micrographs show that the scales of both the blue area of the dorsal wing and the white spots have tightly arranged, parallel ridges (Fig. 2, #1, 2). The ridges of the orange-yellow and red scales are spaced wider than the abovementioned scales, and thus together with the crossribs, they frame rather large windows (Fig. 2, #3–5). The scales of the greenish ventral wing area are particularly widely spaced (Fig. 2, #6).

The transmission electron microscopy results corroborate the different scale architectures. In all the scales, the lower lamina is a thin plate. The blue (Fig. 2a-c, #1) and white (Fig. 2d-f, #2) scales have an upper lamina consisting of tall ridges with characteristic multilayer-forming folds (Fig. 2c, f). The blue scales have pillars below the ridge structures with high electron density, which indicates a substantial amount of melanin (Fig. 2c), quite in contrast with the white scales, which appear to be unpigmented (Fig. 2f). The other scales have the standard structure of butterfly wing scales, where the upper lamina is a loose network of parallel ridges connected by crossribs resting with pillars on the lower lamina.

### Optics of isolated scales

**Fig. 3:**
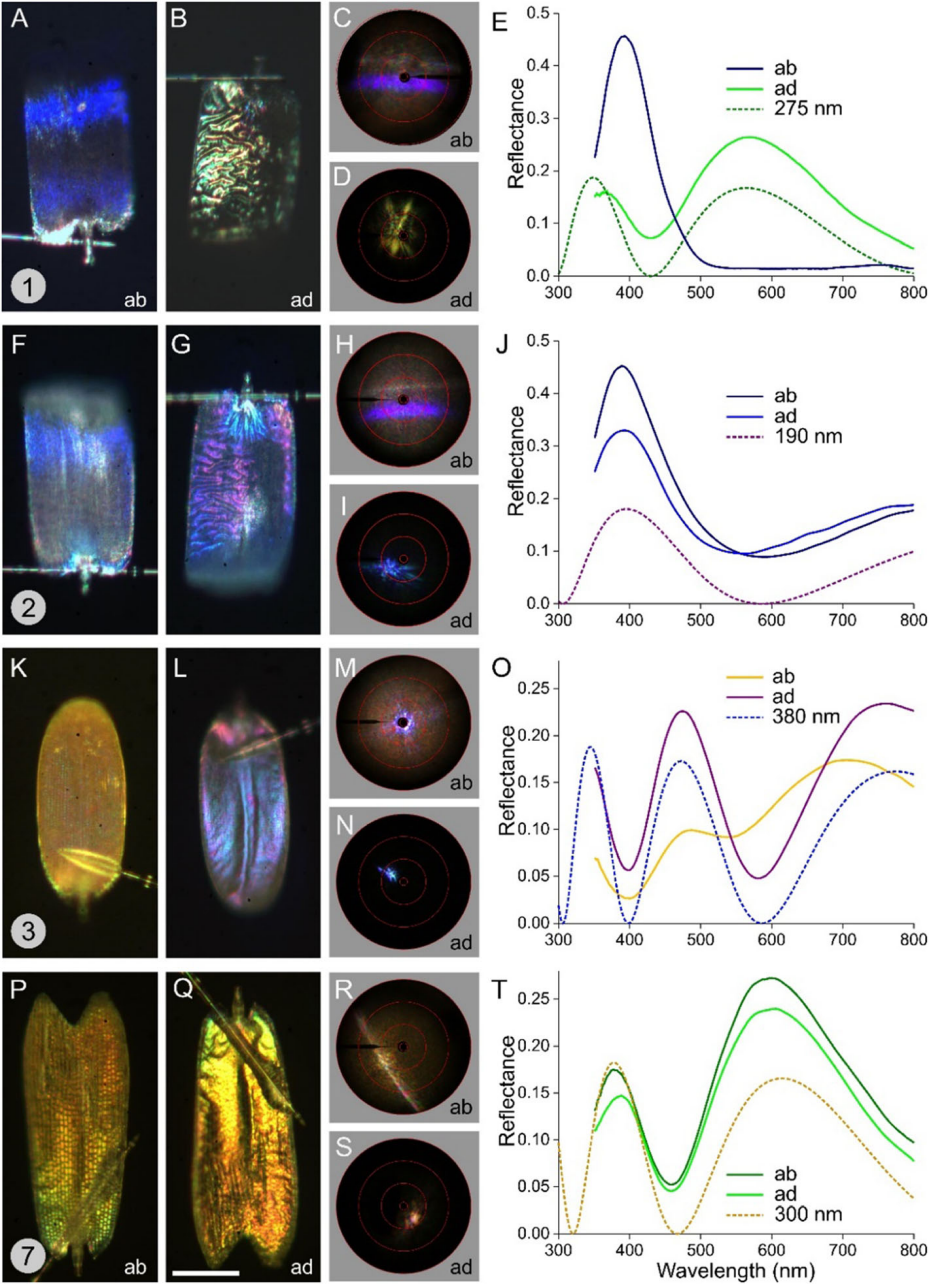
Light micrographs, scatterograms and reflectance spectra of isolated wing scales. **a**-**e** Blue scale. **f**-**j** White scale. **k**-**o** Orange-yellow scale of the dorsal forewing. **p**-**t** Orange-yellow scale of the ventral forewing. **a**, **b**, **f**, **g**, **k**, **l, p**, **q** Epi-illumination light micrographs of the abwing side (1st column) and adwing side (2nd column); scale bar: 50 nm. The numbers 1–3 and 7 in **a**, **f**, **k**, and **p** correspond with the numbered areas in Fig. 1b, e. 3rd column: Scatterograms of the abwing and adwing side; the red circles indicate scattering angles of 5°, 30°, 60°, and 90°. 4th column: Reflectance spectra of the ab- and adwing sides and of chitinous thin films with thicknesses of 275, 190, 380, and 300 nm

The anatomy allows a convenient interpretation of the results of imaging scatterometry and reflectance spectrophotometry that we performed on individual wing scales. We therefore glued the scales to a glass pipette so that the abwing (upper) and adwing (lower) sides of the same scale could be investigated (Fig. 3). Figure 3a shows the abwing side of a scale taken from the blue dorsal wing area. With epi-illumination microscopy, the scale area that reflects blue incident light back into the microscope objective is limited to a band, which occurs because the scale is rather curved and not flat. The scale area outside the blue band is black (Fig. 3a) because the incident light is effectively absorbed by the melanin of the scale ridges and trabeculae.

Very similar to *Morpho* wing scales, the blue reflections are created by the ridges, which consist of a stack of lamellae. Hence, we expected a scatterogram similar to those of *Morpho* scales [24]. Indeed, the abwing (upper) side of the blue scale produces a scatterogram with a distinct blue stripe and a fainter stripe (Fig. 3c). The two stripes in the scatterogram are due to the ridge lamellae of the upper lamina and the thin film of the lower lamina, and the stripes are spatially segregated because the ridge lamellae are inclined with respect to the lower lamina, similar to *Morpho* wing scales [8]. Similar to *Morpho* scales, the abwing reflectance spectrum, measured with a microspectrophotometer, is a narrow band (peak at ~ 400 nm, Fig. 3e). The blue scale’s adwing side has a greenish color and is rather wrinkled (Fig. 3b). As a result, its scatterogram shows a distinctly distributed spot (Fig. 3d). The adwing side has a broadband reflectance spectrum; its peak at 570 nm and shape resemble that of a chitinous thin film with a thickness of 275 nm (Fig. 3e). The color as well as the adwing reflectance spectrum of different blue scales somewhat varies, which is in accordance with the TEM anatomy, yielding a spread in lower lamina thickness of 210–310 nm.

The anatomy showed that a scale from a white spot (Fig. 3f) closely resembles a blue scale with multilayered ridges (Fig. 2c, f). In fact, the white and blue scales feature identical blue bands, demonstrating similar scale curvatures. However, the white scale’s area outside the blue band is whitish because an appreciable fraction of the incident light is backscattered, clearly because of the absence of strongly absorbing melanin pigment. The scatterogram of the abwing side of the white scale (Fig. 3h) closely resembles the blue scale’s abwing scatterogram (Fig. 3c), but there is a background signal due to diffuse scattering by the unpigmented scale. Accordingly, the abwing reflectance spectrum, peaking again at approximately 400 nm, has a substantial background signal. The adwing side of the white scale is blue-purplish (Fig. 3g), and due to its wrinkles, the scatterogram is again slightly spread out (Fig. 3i). As the reflectance spectrum measured locally from the adwing side resembles the abwing spectrum, the spectrum is presumably the result of a lower lamina with a thickness of 190 nm (Fig. 3j). As with the blue scales, the anatomy showed that the lower lamina of the white scales also has a variable thickness, 185–310 nm, which results in a variable adwing reflectance spectrum and color (see Fig. 3g).

An orange-yellow scale of the dorsal forewing (Fig. 3k) has a strongly blue-colored adwing side (Fig. 3l). The scatterogram of the abwing side shows a diffuse pattern due to scattering by the upper lamina in a wide angular space (Fig. 3m). The adwing scatterogram shows a distinct spot because of the locally flat lower lamina, acting as a thin-film reflector (Fig. 3n). The reflectance spectrum of the adwing side indicates that the local thickness of the lower lamina is 380 nm (Fig. 3o). The TEM anatomy of several orange-yellow scales of the dorsal forewing yielded a thickness range of 300–385 nm. The abwing reflectance spectrum, which has peaks and valleys corresponding to those in the adwing spectrum, can be understood as partly the result of blue/purple light reflected by the thin-film lower lamina and yellow light reflected by the pigmented upper lamina (Fig. 3o).

Somewhat surprisingly, a rather similar orange-yellow scale from the ventral forewing (Fig. 3p) features at the adwing side in the proximal area a quite different, strongly yellow metallic reflection (Fig. 3q). The abwing scatterogram shows a clear line due to ridge diffraction plus a diffuse background, representing widespread scattering by the scale structures (Fig. 3r). The adwing scatterogram is dotlike (Fig. 3s) due to the locally rather flat lower lamina (Fig. 3q), which behaves like a thin-film reflector with a thickness of 300 nm (Fig. 3t). The ab- and adwing reflectance spectra are similar, indicating the presence of only a minor amount of pigment.

### The lattice of scales on the wing

**Fig. 4:**
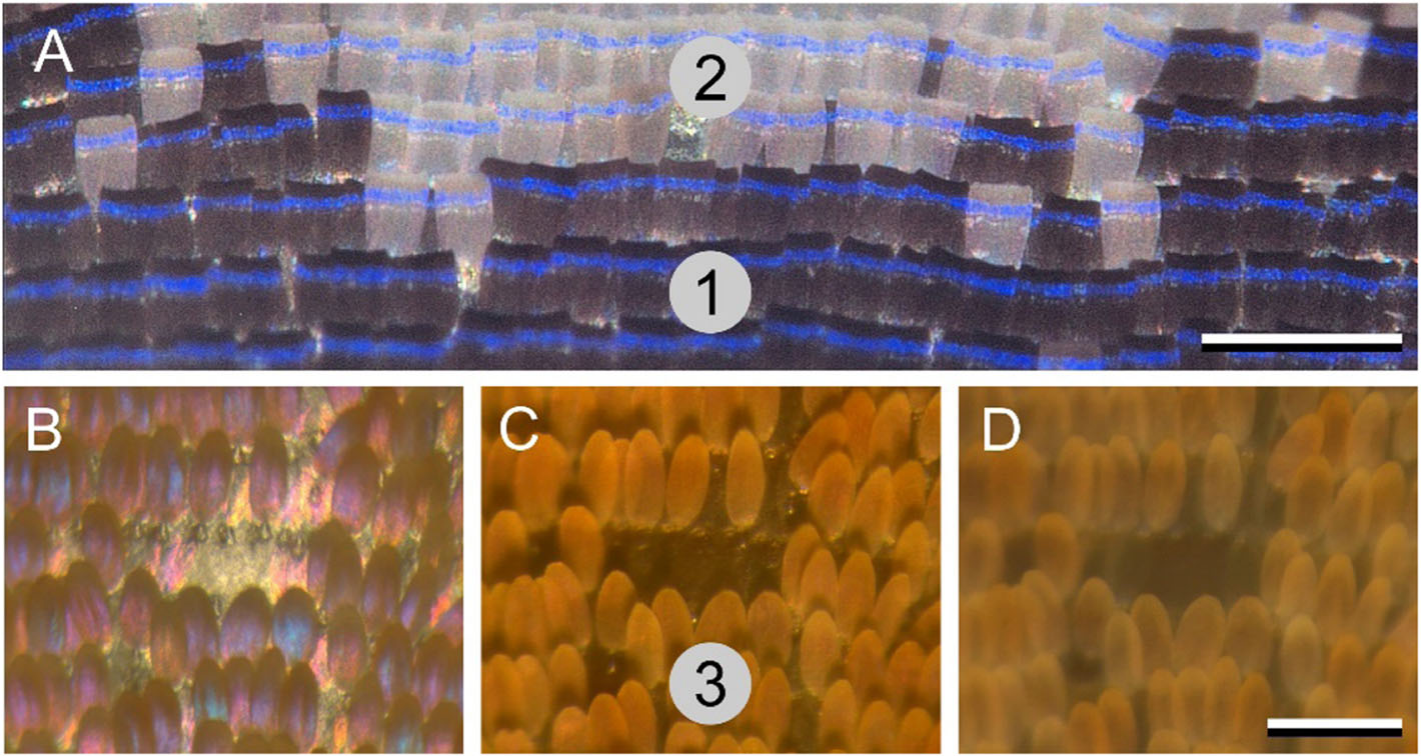
The lattice of scales on the wing. **a** Epi-illumination of the transition area of a white spot in the blue area on the dorsal forewing, showing a similar blue stripe on the light- and dark-colored scales. **b**-**d** Epi-illumination light micrographs of an orange-yellow wing area on the dorsal forewing, with a few missing scales in the center. **b** Normal illumination with linearly polarized light (parallel polarizer and analyzer). **c** Idem with a crossed polarizer and analyzer. **d** Oblique illumination (~ 45°) with unpolarized light. The numbers 1–3 in **a** and **c** correspond to the numbered areas in Fig. 1b, e. Scale bars **a-d**: 200 μm

With the gained insight into the optics of single scales, we can understand the coloration of the butterfly’s wings when covered by the intact lattice of scales. Epi-illumination microscopy of a transition area with blue and white scales on the dorsal forewing demonstrates that both scale types have the same blue band but on a black or white background, respectively (Fig. 4a). When rotating the wing around an axis parallel to the blue bands, the transition area shifts over the scales. In natural conditions, the scales’ curvature is presumably used to radiate the blue color into a large spatial angle. The melanin pigment of the black-blue scales effectively absorbs scattered stray light, creating a contrasting background for the reflection of the blue color (see also Fig. 3e). As the white-blue scales lack pigmentation, part of the incident light is scattered and reflected by the scales as well as by the underlying wing substrate, which thus creates a white background (Fig. 4a). This phenomenon ultimately results in white spots inside the blue areas of the dorsal forewings (Fig. 1b).

A rather different coloration system is encountered in the orange-yellow spots of the dorsal forewing. Figure 4b-d shows an area where part of the scales is removed, revealing the wing substrate. With approximately normal illumination (and normal observation, i.e., perpendicular to the wing), applying linear polarized light and using a parallel analyzer, the wing substrate reflection is white-yellowish, but surprisingly, the scales show a mixture of blue-purplish colors (Fig. 4b). The latter clearly emerges from the thin-film lower lamina (Fig. 3l). With the analyzer in the crossed position, both the specular reflections from the wing substrate and the thin-film reflections of the scales’ lower lamina are extinguished, and the remaining depolarized reflected light has an orange-yellow color (Fig. 4c). A similar color emerges from the scales (but not the wing substrate) when illuminating the same area from an oblique (~ 45°) direction (Fig. 4d). With oblique illumination, only a small fraction of the incident light will be able to enter the scale windows, while a major part of the applied light will hit the scattering ridges and crossribs. As they contain the short-wavelength-absorbing yellow pigment, the color seen with wide-angular, natural illumination is principally determined by the pigmentation.

Close-up inspection of the red scales shows that they behave very similar to the orange-yellow scales. Here, a red pigment causes the overall red color, but with normal illumination, an additional blue flare also exists, which emerges from the scales’ lower lamina, and the same phenomenon can be observed in the brown, melanized scales of the wing margin (not shown; however, see the UV hump in the reflectance spectrum of Fig. 1h, #5).

### Coloring unpigmented scales by thin-film interference

**Fig. 5:**
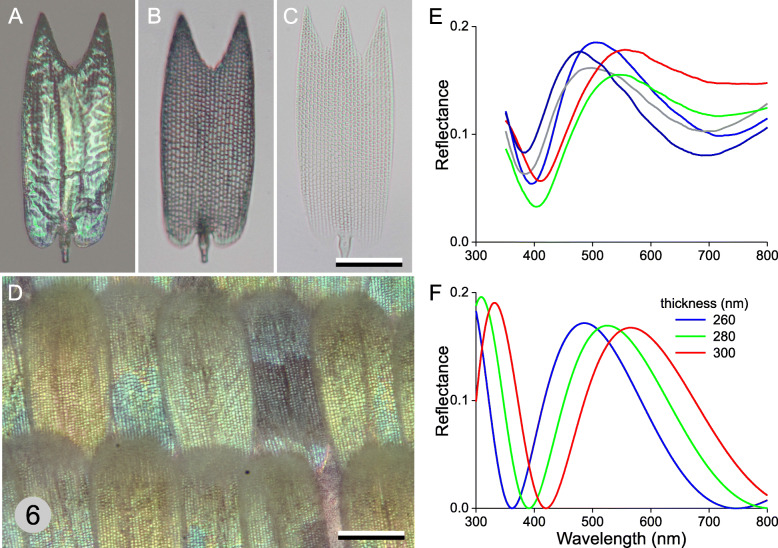
Unpigmented ventral wing scales. **a** Epi-illumination image of the adwing side of an isolated ventral hindwing scale on an object slide. **b** The same scale in transmitted light. **c** Another ventral hindwing scale in oil immersion in transmitted light. **d** Lattice with varying colored scales on the ventral hindwing (Fig. 1b, #6). Scale bars: **a**-**d** 50 μm. **e** Reflectance spectra of a number of adjacent ventral hindwing scales. **f** Reflectance spectra of chitinous thin films with thicknesses of 260, 280, and 300 nm

Similar to the scales in Fig. 3p (#7), the scales on the ventral hindwing have large windows (Fig. 2q, r, #6), and therefore, not only normally but also obliquely incident light will largely pass the ridges and crossribs and thus will subsequently reach the lower lamina unhampered upon reaching the lower lamina. This light will be partly reflected, depending on the thickness of the lower lamina. The lower lamina of the ventral hindwing scales is again quite wrinkled, although epi-illumination still produces a distinct color due to light interference in the thin film (Fig. 5a). Applying transmitted light at an isolated scale on a microscope slide may suggest the presence of melanin (Fig. 5b), but in immersion oil, the scales become almost invisible, meaning that the pigmentation is negligible (Fig. 5c). The thin-film characteristics of the lower lamina of an unpigmented scale therefore play a principal role in the scale coloration.

A local set of ventral wing scales observed in situ on the wing appears to be somewhat variably colored, indicating that the thickness of the lower lamina is not constant (Fig. 5d). Indeed, reflectance spectra measured from a number of adjacent scales vary slightly (Fig. 5e), similar to the reflectance spectra of chitinous thin films with thicknesses varying between 260 and 300 nm (Fig. 5f). The experimentally obtained spectra have a background signal, whereas the theoretical spectra show zero reflectance at specific wavelengths. The background signal is partly due to scattering contributions by the ridges and crossribs, but additional in situ back-scattering from the wing substrate is also important.

## Discussion

A considerable variety of coloration methods are employed by *S. charonda* to realize its rich patterning. First, stacks of lamellae in the ridges create the large blue iridescent areas of the dorsal wings [11–13]. The white spots also display the same iridescence, but it is largely drowned by the white scattering resulting from the absence of pigment. The universally occurring brown wing margins are due to melanin pigment, and the local presence of yellow and red pigment is also apparent.

The pigments extracted from the orange-yellow and red scales yielded absorbance spectra peaking at 380 and 470 nm, respectively (Fig. 1i). Pigments extracted from the yellow and red scales of various *Heliconius* species had similar absorption peak wavelengths [32] and were identified as 3-OH-kynurenine and ommochrome, the characteristic pigments of nymphalids [10]. However, for the *Heliconius* ommochrome, the ratio of the absorption in the UV and blue-green spectra is considerably less than that of the pigment of *Sasakia*’s red scales, which suggests that these scales contain a considerable amount of 3-OH-kynurenine in addition to the ommochrome. Similarly, the orange-yellow scales may have variable traces of ommochrome in addition to the substantial amount of 3-OH-kynurenine.

Interestingly, the reflectance spectrum of the red scales (Fig. 1h, #4) shows a dip at ~ 720 nm, which is quite pronounced in the reflectance spectra of the red wing areas of *Heliconius* species (Figs. 1c, 2d of [32]). The dip suggests the presence of an as yet unidentified pigment, but alternatively, the ommochromes may have a minor long-wavelength absorption band.

The scatterograms and reflectance spectra of the adwing side of the investigated scales demonstrate that the lower lamina acts as a thin-film reflector. The lower lamina thus plays an active role in the scale coloration because light incident from the abwing side is always only partly reflected and absorbed, and the transmitted light reaching the lower lamina will be partly reflected there and will then contribute to the total scale reflectance. We find that this phenomenon is most prominently the case in unpigmented scales of the ventral hindwings of *S. charonda*, at least in the Japanese subspecies *S. c. charonda*. In other subspecies, e.g., *S. c. coreana* of Korea, the scales of the ventral hindwings are sometimes slightly pale, yielding a less dark color. As the wings are closed when resting, the ventral wing coloration presumably functions as camouflage, which may indicate that the resting habitats of the various subspecies differ. At any rate, the thickness of the lower lamina of the scales plays an important role in the resulting wing coloration, which is probably subject to selection and genetic control [26, 30].

The optical principles governing the coloration of *S. charonda* are generally recognizable in other nymphalid butterflies. Very similar coloration patterns are displayed by the related purple emperor butterflies *Apatura ilia* and *A. iris* [1, 20, 21]. The multilayered ridges creating the blue iridescence of the dorsal wings are similar to those causing the strikingly blue color of *Morpho* butterflies. This stacking of blue unpigmented scales creating white wing spots has been previously recognized in nymphalids where the blue scales are the result of a blue-reflecting lower lamina. Orange-yellow and red pigmentary colors are commonly observed in many other nymphalids. The role of the lower lamina in providing colors other than blue, as occurs in the yellow/greenish wings of the ventral hindwing of the emperors, for instance, has so far remained underilluminated. We may conclude that the male *S. charonda*, by applying a wide gamut of optical techniques, clearly succeed in donning a bright and colorful dorsal wing cover, which is effective in courtship, and a ventral wing pattern, which is suitable for camouflage.

## Conclusions

The impressively rich wing coloration of the male *Sasakia charonda* appears to be realized by a combination of structural and pigmentary coloration methods, which have been elucidated by applying various optical and anatomical approaches. Multilayered ridges in the scales on the dorsal wings create a striking blue iridescence. The scales’ lower lamina, acting as a thin-film reflector, generally plays an important role, particularly in coloring the ventral wings. The common nymphalid pigments, 3-OH-kynurenine and ommochrome, as well as melanin, add to the diverse coloration palette.

## Data Availability

The datasets supporting the conclusions of this article are included within the article.

## References

[1] Ćurčić SB, Pantelić DV, Ćurčić BPM, Savić-Šević SN, Makarov SE, Lačković VB, Labudović-Borović MM, Ćurčić N, Stojanović DV (2012). Micro- and nanostructures of iridescent wing scales in purple emperor butterflies (Lepidoptera: *Apatura ilia* and *A. iris*). Microsc Res Tech.

[2] Descimon H (1971). Les ptérines des *Pieridae (Lepidoptera)* et leur biosynthèse. I - Identification des principales ptérines de *Colias croceus* (Fourcroy) et de quelques autres espèces de *Pieridae*. Biochimie.

[3] Eguchi E, Meyer-Rochow VB (1983). Ultraviolet photography of forty-three species of Lepidoptera representing ten families. Annot Zool Jpn.

[4] Figon F, Casas J (2019). Ommochromes in invertebrates: biochemistry and cell biology. Biol Rev.

[5] Ghiradella H (2010). Insect cuticular surface modifications: scales and other structural formations. Adv Insect Physiol.

[6] Ghiradella H, Locke M (1998). Hairs, bristles, and scales. Microscopic anatomy of invertebrates, Vol 11A: Insecta.

[7] Ghiradella H (1984). Structure of iridescent lepidopteran scales: variations on several themes. Ann Entomol Soc Am.

[8] Giraldo MA, Stavenga DG (2016). Brilliant iridescence of *Morpho* butterfly wing scales is due to both a thin film lower lamina and a multilayered upper lamina. J Comp Physiol A.

[9] Kinoshita M, Sato M, Arikawa K (1997). Spectral receptors of nymphalid butterflies. Naturwissenschaften.

[10] Koch PB (1993). Production of [14C]-labeled 3-hydroxy-L-kynurenine in a butterfly, *Heliconius charitonia* L.(Heliconidae), and precursor studies in butterfly wing ommatins. Pigment Cell Res.

[11] Matĕjková-Plšková J, Shiojiri S, Shiojiri M (2009). Fine structures of wing scales in *Sasakia charonda* butterflies as photonic crystals. J Microsc.

[12] Matĕjková-Plšková J, Jancik D, Mašlá M, Shiojiri S, Shiojiri M (2010). Photonic crystal structure of wing scales in *Sasakia charonda* butterflies. Mat Transact.

[13] Medina JM, Díaz JA, Valero EM, Nieves JL, Vukusic P (2014). Detailed experimental characterization of reflectance spectra of *Sasakia charonda* butterfly using multispectral optical imaging. Opt Eng.

[14] Nijhout HF (1991). The development and evolution of butterfly wing patterns.

[15] Nijhout HF (1997). Ommochrome pigmentation of the *linea* and *rosa* seasonal forms of *Precis coenia* (Lepidoptera: Nymphalidae). Arch Insect Biochem Physiol.

[16] Nijhout HF (2010). Molecular and physiological basis of colour pattern formation. Adv Insect Physiol.

[17] Nishikawa H, Iga M, Yamaguchi J, Saito K, Kataoka H, Suzuki Y, Sugano S, Fujiwara H (2013). Molecular basis of wing coloration in a Batesian mimic butterfly, *Papilio polytes*. Sci Rep.

[18] Onslow H (1923). On a periodic structure in many insect scales, and the cause of their iridescent colours. Phil Trans R Soc Lond B.

[19] Panettieri S, Gjinaj E, John G, Lohman DJ (2018). Different ommochrome pigment mixtures enable sexually dimorphic Batesian mimicry in disjunct populations of the common palmfly butterfly, *Elymnias hypermnestra*. PLoS One.

[20] Pantelić D, Ćurčić S, Savić-Šević S, Korać A, Kovačević A, Ćurčić B, Bokić B (2011). High angular and spectral selectivity of purple emperor (Lepidoptera: *Apatura iris* and *A. ilia*) butterfly wings. Opt Express.

[21] Schenk F, Stavenga DG. The lesser purple emperor butterfly, *Apatura ilia*: from mimesis to biomimetics. Faraday Discuss, in press. 2020. 10.1039/D0FD00036A. 10.1039/d0fd00036a 32760964

[22] Stavenga DG (2014). Thin film and multilayer optics cause structural colors of many insects and birds. Mat Today Proc.

[23] Stavenga DG, Leertouwer HL, Meglič A, Drašlar K, Wehling MF, Pirih P, Belušič G (2018). Classical lepidopteran wing scale colouration in the giant butterfly-moth *Paysandisia archon*. PeerJ.

[24] Stavenga DG, Leertouwer HL, Pirih P, Wehling MF (2009). Imaging scatterometry of butterfly wing scales. Opt Express.

[25] Stavenga DG, Leertouwer HL, Wilts BD (2014). Coloration principles of nymphaline butterflies - thin films, melanin, ommochromes and wing scale stacking. J Exp Biol.

[26] Thayer RC, Allen FI, Patel NH (2020). Structural color in *Junonia* butterflies evolves by tuning scale lamina thickness. Elife.

[27] Umebachi Y (1985). Papiliochrome, a new pigment group of butterfly. Zool Sci.

[28] Vukusic P, Sambles JR (2003). Photonic structures in biology. Nature.

[29] Vukusic P, Sambles JR, Lawrence CR (2004). Structurally assisted blackness in butterfly scales. Proc R Soc B.

[30] Wasik BR, Liew SF, Lilien DA, Dinwiddie AJ, Noh H, Cao H, Monteiro A (2014). Artificial selection for structural color on butterfly wings and comparison with natural evolution. Proc Natl Acad Sci U S A.

[31] Wijnen B, Leertouwer HL, Stavenga DG (2007). Colors and pterin pigmentation of pierid butterfly wings. J Insect Physiol.

[32] Wilts BD, Vey AJ, Briscoe AD, Stavenga DG (2017). Longwing (*Heliconius*) butterflies combine a restricted set of pigmentary and structural coloration mechanisms. BMC Evol Biol.

[33] Wilts BD, IJbema N, Stavenga DG (2014). Pigmentary and photonic coloration mechanisms reveal taxonomic relationships of the Cattlehearts (Lepidoptera: Papilionidae: *Parides*). BMC Evol Biol.

